# Adopting workload-based staffing norms at public sector health facilities in Bangladesh: evidence from two districts

**DOI:** 10.1186/s12960-021-00697-7

**Published:** 2022-01-28

**Authors:** Md Nuruzzaman, Tomas Zapata, Valeria De Oliveira Cruz, Sabina Alam, Samiun Nazrin Bente Kamal Tune, Taufique Joarder

**Affiliations:** 1 Human Resources for Health, WHO Bangladesh, Dhaka, Bangladesh; 2 Human Resources for Health, WHO South-East Asia Regional Office, Delhi, India; 3 Health Financing and Governance, WHO South-East Asia Regional Office, Delhi, India; 4 Health Services Division, Ministry of Health and Family Welfare, Dhaka, Bangladesh; 5 BRAC James P Grant School of Public Health, BRAC University, Dhaka, Bangladesh; 6 Public Health Foundation of Bangladesh, Dhaka, Bangladesh

**Keywords:** Workload, Bangladesh, Staffing norms, Human resources for health

## Abstract

**Background:**

Bangladesh’s Health system is characterized by severe shortage and unequitable distribution of the formally trained health workforce. In this context, government of Bangladesh uses fixed staffing norms for its health facilities. These norms do not always reflect the actual requirement in reality. This study was conducted in public sector health facilities in two selected districts to assess the existing staffing norms with the purpose of adopting better norms and a more efficient utilization of the existing workforce.

**Methods:**

To carry out this assessment, WHO’s Workload Indicators of Staffing Need (WISN) method was applied. Selection of the two districts out of 64 and a total of 24 health facilities were made in consultation with the formally established steering committee of the Ministry of Health. Health facilities, which were performing well in serving the patients during 2016–2017, were selected. This assessment examined staffing requirement of 20 staff categories.

**Results:**

Based on the computer-generated WISN results, most of the staff categories were found to have a workload pressure of Very High (seven out of 20 staff categories), followed by Extremely High (five staff categories). Two staff categories had high, three had moderately high, two normal, and one low workload. Nurses were found to be predominantly occupied with support activities (50–60% of working time), instead of actual nursing care. Regarding vacancy, if all the vacant posts were filled, understandably, the workload would reduce, but not yet sufficient to meet the existing staff requirements such as consultants, general physicians and nurses at the district and sub-district/upazila-based hospitals.

**Conclusion:**

The existing staffing norms fall short of the WISN staffing requirement. The results provide evidence to prompt a revisit of the staffing policies and adopt workload-based norms. This can be supplemented by reviewing the scope of practice of the staff categories in their respective health facilities. In the short term, government might consider redistributing existing workforce as per workload. In the long term, revision of staffing norms is needed to provide quality health services for all.

## Background

Bangladesh was identified as one of the severe health workforce shortage countries by the World Health Organization (WHO) in the World Health Report 2006 [1]. This was because the country had a density of doctors, nurses and midwives of 5.8 per 10,000 population in 2006 [2]. The WHO Global Strategy on Human Resources for Health: Workforce 2030 estimated an indicative requirement of the threshold density of 44.5 doctors, nurses and midwives per 10,000 population to achieve SDGs by 2030 [3]. According to the latest WHO South-East Asia Regional Office assessment of health workforce in SEAR countries, Bangladesh has reached a density of doctor, nurse and midwife of 9.9 [4]. This indicates that the country has a chronic shortage of the number of formally trained of health workforce. Besides, health services in Bangladesh are crippled by several other health workforce challenges such as maldistribution between rural and urban areas, lack of career development opportunities, weak knowledge base, high vacancy rate in the public sector and poor working conditions [5, 6].

In this context, the Government of Bangladesh (GOB) adopted the Essential Services Package (ESP) as a strategy in 2016–2017 towards achieving Universal Health Coverage [7]. ESP was designed to deliver health services at the facilities located at district level and below to household level under the Ministry of Health and Family Welfare (MOHFW). Now, to guide health workforce recruitment and salary budget and to determine staffing level at those public-sector health facilities, MOHFW uses the fixed staffing norms established in 2008, approved by the Ministry of Public Administration [8]. These staffing norms vary according to the health facility type and availability of healthcare services. There is little scope to account for workload variation of staffs, or facility-specific outputs, leading to inefficiency. The fixed staffing norms adopted by the MOHFW are based on ‘bed number’ and ‘number of population to serve’ and these are not responsive to the factors like population growth, geographical location and climate change, physical facilities availability, changing pattern of the disease burden and patient preferences [9].

In this regard, the Bangladesh Health Workforce Strategy-2015 urges determination of health workforce need, and project demand up to 2030 as per level of care (i.e., primary, secondary and tertiary levels) [10]. To do such, the strategy recommends consideration of workload in determining the staffing needs of different health facilities.

In order to assist Member States to assess workload of the staff, the WHO developed the “Workload Indicators of Staffing Need (WISN)” in 1998 and introduced its computerized version in 2010 [11, 12]. This article describes the application of the WISN method in two selected districts in Bangladesh and how staffing norms can be reshaped in Bangladesh. The main purpose is to explain the limitations of the currently practiced staffing norms in different health facilities in a district health system and to generate evidence to inform policy for appropriate staffing decision and staff deployment for more efficient use of the scarce human resources.

## Methods

This study was initiated by Human Resources Development (HRD) Unit, MOHFW with reference to the Bangladesh Health Workforce Strategy-2015. This initiative was technically supported by the World Health Organization (WHO) Bangladesh and data collection and analysis was carried out by the BRAC James P Grant School of Public Health, BRAC University. A national-level WISN training of trainers including district managers from the two pilot districts was conducted by WHO in November 2016.

In order to assess the current workload of the existing staff at their respective health facilities during the study period, WHO’s WISN manual was adopted and followed. According to this manual, there are eight steps to apply the WISN method: (1) determining priority cadres and health facility types; (2) estimating available working time; (3) defining workload components; (4) setting activity standards; (5) establishing standard workloads; (6) calculating allowance factors; (7) determining staff requirement based on WISN, and (8) analyzing and interpreting WISN results. All those steps were carried out in consultation and collaboration with the Steering Committee (SC) established at national level, Technical Taskforce (TT) and Expert Working Groups (EWGs). The SC was established by the MOHFW, GOB and provided overall guidance to the whole initiative. The TT led the implementation of the project and EWGs were formed to provide expert opinion on determination of service activity standard based on ESP components.

This assessment was conducted in two selected districts out of 64 in Bangladesh during July to November 2017. Table 1 shows composition of district health system, its type, catchment population, major essential services delivered, key staff and staffing norms with reference to the 2008 staffing standard of the GOB.

**Table 1:** District healthcare system of Bangladesh [13]

Type of health facility	Location (catchment population size)	Major ESP delivered	Staffing norms and key staff numbers with reference to the 2008 staffing standard
District-based General Hospital (250-bed)	District town (2,500,000)	In-patient and out-patient services Maternal and child health care Orthopedic services Minor to moderate level surgical services Common consultant/specialized services Medical imaging and laboratory services	Staffing norms is based on the total bed size of the hospital Consultant (22) Medical Officers/General Physicians (23) Nursing Staff (80)
Maternal and Child Welfare Center (10-bed hospital)	District town (2,500,000)	Maternal and child health services including normal delivery and caesarian section Family planning services In-patient and out-patient services	Staffing norms is based on the total bed size Medical Officers (MO) (2) Family Welfare Visitor (2)
Upazila or Sub-District Health Complex (50-bed hospital)	Upazila/Sub-District town (250,000–400,000)	In-patient and out-patient services Maternal and child health care Orthopedic cares Minor surgical services Major consultant/ specialized services	Staffing norms based on the total bed numbers Consultant (10) Medical officer (7) Nursing Staff (15) Sub-Assistant Community Medical Officer (1)
Union Health and Family Welfare Center (only outdoor)	Union-level- rural periphery (40,000)	Out-patient services Maternal and child health care Family planning services	Ad hoc norms Family Welfare Visitor (1) Sub-Assistant Community Medical Officer (1)
Union Sub-Center (only outdoor)	Union-level- rural periphery (40,000)	Out-patient services Maternal and child health care	Ad hoc norms Medical officer (1) Sub-Assistant Community Medical Officer (1)
Community Clinic (only outdoor)	Ward level (6000)	Out-patient services Maternal and child health care Family planning services	Population-based staff norms (i.e., one staff per 6000 population) Community Health Care Provider (1) Health Assistant (1) Family Welfare Assistant (1)

The bulk of primary health care services are delivered through Community Clinics (CCs), Union Sub-Centers (USC), Union Health and Family Welfare Centers (UHFWC) and Upazila Health Complexes (UpHCs). District-based general hospitals provide secondary-level health care and also serves as the referral point for the primary level health facilities. The MOHFW has the primary responsibility for human resource investments of all primary and secondary-level health facilities, whereas the Ministry of Public Administration serves as the approving authority on HR matters (e.g., recruitment rules, post creations, job descriptions, table of organizations and equipment, etc.) on behalf of the government [14].

### Selection of health workforce categories

In consultation with the Steering Committee and Technical Taskforce members and taking consideration of similar studies conducted in other countries, the following 20 categories of health workforce were identified from the selected health facilities (Table 2).

**Table 2:** Facility types and staff categories for the WISN study

Type of facility (*N* = 24)	Study sites	Staff category
District hospital (*n* = 2)	Jhenaidah District Hospital (100 bed) and Moulvibazar 250-bed District Hospital	Consultants (Surgery, Anesthesiology, Obstetrics and Gynecology, Ear Nose and Throat, Medicine, Pediatrics, Cardiology, Orthopedics) General Physicians (Medical Officer), Emergency Medical Officer, Resident Medical Officer) Nursing Staff (Senior Staff Nurse, Nursing Supervisor)
Maternal and Child Welfare Center (*n* = 2)	Jhenaidah and Moulvibazar	Medical Officer (MO) Family Welfare Visitor (FWV)
Upazila Health Complex (UpHC) (*n* = 4)	Shailkupa UpHC and Kotchandpur UpHC from Jhenaidah Kulaura UpHC, and Sreemangal UpHC from Moulvibazar	General Physicians, Nursing Staff (Senior Staff Nurse, Nursing Supervisor) Sub-Assistant Community Medical Officer (SACMO)
Union Sub-Center (*n* = 4) and Union Health and Family Welfare Center (*n* = 4)	Jhenaidah: Kacherkhol, Sabdalpur, Moharajpur and Dudhshar, From Moulvibazar: Prithempasha, Bhunabir, Chandnighat and Ashidron	SACMO, FWV
Community clinic (*n* = 8)	From Jhenaidah: Khandakbariya, Kagmari, Kulbaria, and Korotipara, Sripur, From Moulvibazar: Igragaon, Dakkhin Chamatkar, and Akbarpur	Community Health Care Provider Family Welfare Assistant

This assessment put focus on the above categories of health workforce as they have been the leading frontline health care providers

### Defining workload component

According to the WHO’s WISN manual (2010), there are three workload components, which are: (1) health service activity; (2) support activity, and (3) additional activity. Health service activity refers to all the activities performed by all members of the health workforce categories and for which annual statistics are regularly collected, e.g., antenatal care. Support activity supports the implementation of health service activities. These activities are performed by all members of the health workforce categories, but for which annual statistics are not regularly collected, e.g., monthly report writing. Additional activities are those activities, which are performed only by certain (not all) members of the staff, e.g., supervisory role. Annual statistics are not regularly collected for them.

### Study design, sites and sample selection

Since the WISN method was not in practice in the public sector health facilities in Bangladesh, an exploratory study involving both qualitative and quantitative methods was commissioned. The qualitative methods involved document reviews, key informant interviews (KIIs) with policy level persons (mostly from among steering committee and expert group members, total 20), in-depth interviews (IDIs) with the health workforce (total 87, e.g., physicians, nurses, etc.), and observations.

KII respondents were selected on the principles of purposive sampling [15], supplemented by snowball sampling (i.e., based on the reference or suggestion from the key informants). IDI respondents were selected through purposive sampling, based on the respondent’s seniority (assuming better knowledge of the activity standards) and designation (e.g., Medical Doctors, Nursing Supervisors, etc.). Semi-structured guidelines or checklists for document reviews, KIIs, IDIs, and observation were developed and pre-tested in a district-based general hospital near the capital city, Dhaka, before applying for actual data collection. The pre-testing exercise was followed by the training of the data collectors.

The Steering Committee selected the two districts based on selected performance indicators, i.e., low maternal mortality ratio, low child mortality rate and low morbidity rate prevailing in those regions. According to the Health Bulletin 2016, these two districts turned out as the good performing ones, based on the indicators mentioned above [16]. Among the two districts, Jhenaidah is located in south-western part of Bangladesh, under Khulna Division; while Moulvibazar is located in north-eastern part of Bangladesh, under Sylhet Division.

From each district, based on the guidance from steering committee, one District Hospital (DH), one Maternal and Child Welfare Center (MCWC), two UpHCs, two USCs, two UHFWCs, and four CCs were included in this study (Table 2).

The quantitative method involved a time-motion study with all consenting staff during the study period. Each staff was observed twice for a 45-min time, during the first and second half of the day, to minimize the bias due to patient load at different parts of the day. The time-motion data were used only to guide the activity standards proposed in the IDIs.

### Data collection strategy

A central WISN technical taskforce was established and they were technically assisted by the lead author. The principal investigator formed two data collection teams, who were led by field supervisors and sent to the two districts, respectively. Two assigned Investigators were guiding and monitoring them for a sound data collection process. In order to ensure data quality, the principal investigator, co-investigators, WHO team consisting of national and international technical experts, and representatives from the MOHFW conducted field visits to each study district and the health facilities therein.

### Data management and analysis

Based on the WISN manual, the first analytic step was to estimate available working time (AWT) of the staff. This is the time a health worker has available in one year to do his or her work, taking into account authorized and unauthorized absences [11]. For all categories of staff, a uniform number of weeks per year (52 weeks), working days in 1 week (6 days), possible working days in 1 year (52 × 6 = 312 days) were estimated. Next, absent days, such as public holidays (20 days), earned leave (average for each staff category, based on Health Management Information System data), and casual leave (20 days) were deducted to obtain the annual working time in days. Multiplying this with daily working hours (6 h per day), annual working time was calculated in hours.

Based on the current number of staff posted to their respective health facilities, both the difference (current number of staff—required number of staff by WISN), and the ratio (current number of staff/required number of staff by WISN) were calculated. The WISN difference indicates whether the health facilities are relatively understaffed (i.e., when the WISN Difference is negative), overstaffed (i.e., when the WISN difference is positive), or balanced (i.e., when the WISN difference is zero). The WISN ratio indicates whether the staff are experiencing high workload (i.e., when the WISN ratio is lower than one), low workload (i.e., when the WISN ratio is higher than one), or normal workload (i.e., when the WISN ratio is equal to one). For this calculation, the current number of staff of each facility was obtained from the existing human resource information system and later was verified with the head of the facility and its payroll system.

For descriptive purposes, the workload pressure is categorized as Extremely High (WISN ratio between 0.10 and 0.29), Very High (WISN ratio between 0.30 and 0.49), High (WISN ratio between 0.50 and 0.69), Moderately High (WISN ratio between 0.70 and 0.89), Normal (WISN ratio between 0.90 and 1.19), and Low (WISN ratio greater than or equal to 1.20) [11].

## Results

### General WISN findings across district, upazila (sub-district), union and community levels

At an aggregate level (i.e., considering the average required number and WISN ratio across the same types of health facilities), with reference to Table 3, a good number of the staff categories were found to have a workload pressure of Very High (seven out of 20 staff categories), followed by Extremely High (five staff categories). Two staff categories had high, three had moderately high, two normal, and one low workload (Table 3). The highest workload was observed among the following staff categories: Consultants of Medicine (WISN ratio 0.16), Pediatrics (WISN ratio 0.20), Anesthesiology (WISN ratio 0.20), Obstetrics and Gynecology (WISN ratio 0.25), and Surgery (WISN ratio 0.28) (Table 3).

**Table 3:** Analysis of WISN results at aggregate level (average required number and WISN ratio across same types of health facilities)

Staff category	Required staff to cope with the workload	Average number of existing staff	Deficit of staff	Average WISN ratio	Workload pressure
Consultant (Surgery)	7.42	2.0	5.42	0.28	Extremely high
Consultant (Anesthesiology)	4.98	1.0	3.98	0.2	Extremely high
Consultant (Obstetrics and gynecology)	11.0	2.0	9	0.25	Extremely high
Consultant (Orthopedics)	5.04	2.0	3.04	0.41	Very high
Consultant (Ear, Nose and Throat)	2.82	1.5	1.32	0.67	High
Consultant (Medicine)	12.0	2.0	10	0.16	Extremely high
Consultant (Pediatrics)	7.64	1.5	6.14	0.2	Extremely high
Consultant (Cardiology)	4.21	1.5	2.71	0.35	Very high
District Hospital General Physician	35.10	11.0	24.1	0.32	Very high
District Hospital Nurse	135.92	66.0	69.92	0.49	Very high
Maternal and Child Welfare Center (MCWC)—General Physician	4.22	2.0	2.22	0.48	Very high
MCWC Family Welfare Visitor (FWV)	5.8	4.5	1.3	0.81	Moderately high
Upazila Health Complex (UpHC) General Physician	10.59	4.5	6.09	0.43	Very high
UpHC Nurse	18.86	12.7	6.11	0.69	High
UpHC SACMO*	10.43	7.75	2.68	0.75	Moderately high
Union Sub-Center SACMO*	2.33	1.00	1.33	0.49	Very high
UHFWC** SACMO*	1.04	1.00	0.04	1.29	Low
UHFWC FWV	1.18	1.00	0.18	0.85	Moderately high
Community Clinic—Community healthcare provider	1.34	1.0	0.34	0.92	Normal
Community Clinic—Family Welfare Assistant	0.96	1.0	− 0.04	1.1	Normal

*SACMO Sub-Assistant Community Medical officer, *UHFWC* **Union Health and Family Welfare Center

In general, the highest number of health workforce is required (discounting the existing staff) for DH Nursing Staff (135.92 on an average), followed by DH General Physicians (35.10 on an average), and UpHC Nursing Staff (18.86) (Table 3).

Highest shortage (i.e., deducting required number from current number of staff) is observed among the Consultant (Obstetrics and Gynecology) category of Jhenaidah District Hospital (Difference − 14.63), followed by Consultant (Medicine) in both the District Hospital (Difference − 11.07 in Jhenaidah, and 12.94 in Moulvibazar). Although workload pressure is relatively lower (WISN Ratio 0.49 and 0.48 in Jhenaidah and Moulvibazar, respectively), the shortage of District Hospital Nursing Staff (i.e., Senior Staff Nurse and Nursing Supervisor) is the highest (WISN Difference − 64.86 and − 74.98 in Jhenaidah and Moulvibazar, respectively) among all the staff categories. This is followed by that among the General Physicians in District Hospitals (WISN Difference − 23.29 and − 24.90 in Jhenaidah and Moulvibazar, respectively).

Normal or low workload is very uncommon, except for UHFWC SACMOs, CC CHCPs and CC FWAs (average WISN ratio 1.29, 0.92, and 1.1, respectively). Excess staff and low workload pressure is observed particularly among CHCPs in four CCs (out of eight): Khandakbaria (WISN Difference 0.11 and ratio 1.12), Kulbaria (WISN difference 0.23 and ratio 1.37), Dakkhin Chamatkar (WISN difference 0.12 and ratio 1.14), and Akbarpur (WISN difference 0.14 and ratio 1.16); and FWAs in four CCs (out of five): Khandakbaria (WISN difference 0.25 and ratio 1.33), Korotipara (WISN difference 0.15 and ratio 1.18), Sripur (WISN difference 0.04 and ratio 1.04), and Dakkhin Chamatkar (WISN difference 0.21 and ratio 1.27).

### Workload components-wise workload pressures

**Fig. 1:**
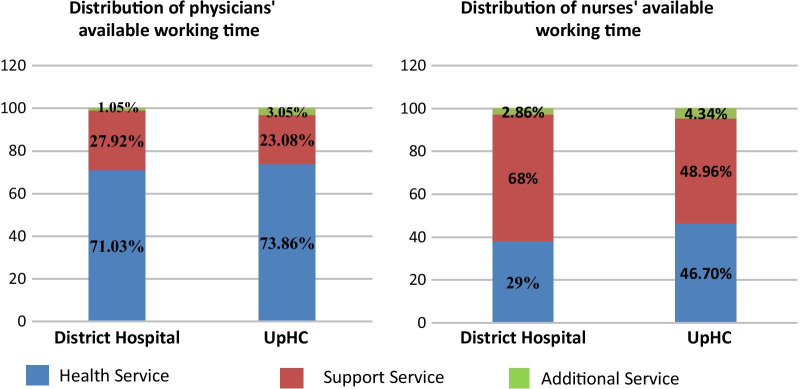
Proportion of available working time of physicians and nurses spent for health service, support service and additional activities

Physicians of Jhenaidah and Moulvibazar District hospitals and Upazila Health Complexes (UpHCs) spend 27–29% of their AWT on support and additional activities like meetings, medico-legal cases, national day celebration, supervision of clinical staffs, etc. Nursing staffs of Jhenaidah and Moulvibazar District hospital and Upazila level spend more than half (53–62%) of the AWT on support services and additional services, such as making patient beds, linen management, maintenance of stock register, cleaning supervision, etc. They provide health services only on 38–47% of their AWT time (Fig. 1).

### Change of workload if vacancies are filled against the number of sanctioned posts

“Sanctioned post” refers to the post and its designation to which staff is assigned in a particular public-sector health facility as per the national staffing norms. The post and its number are approved by the MOPA and Ministry of Finance. Each public sector health facility has its own organogram and the organogram reflects the number of sanctioned posts including designation. If the vacant posts are filled, understandably, the workload is reduced. However, only filling up the vacant posts are not enough in the case of some of the staff categories, such as general physicians and nurses at the DH (Table 4).

**Table 4:** Change of workload if vacancies of physician and nursing posts are filled

Health facility	Staff category	Current number of staff	Required number, based on WISN	WISN ratio	Sanctioned number of staff [8]	WISN ratio as per sanctioned number of staff
Jhenaidah DH	General Physician	11	34.29	0.32	17	0.50
	Nursing Staff	62	126.86	0.49	80	0.63
Moulvibazar DH	General Physician	11	35.9	0.31	22	0.61
	Nursing Staff	70	144.98	0.48	80	0.55
Shailkupa UpHC	General Physician	4	8.14	0.49	7	0.88
	Nursing Staff	14	16.08	0.87	15	0.94
Kotchandpur UpHC	General Physician	3	10.71	0.28	7	0.64
	Nursing Staff	15	22.8	0.66	15	0.65
Kulaura UpHC	General Physician	4	12.28	0.33	7	0.58
	Nursing Staff	10	16.08	0.62	15	0.93
Sreemangal UpHC	General Physician	7	11.23	0.62	7	0.64
	Nursing Staff	12	20.46	0.59	15	0.75

The WISN ratios were calculated based on the workload of the current number of staff. These ratios help to determine the most appropriate minimum staffing for each level of health facility to provide the expected range of services. Table 4 compares the WISN-based staff requirement with the existing staffing norms. For example, during the study period, Jhenaidah district hospital had only 11 general physicians, but workload-based requirement was 34. Within the existing staffing norms, government could recruit only 17 physicians [8]. Same scenarios were observed for both doctor and nurse categories in all DHs and UpHCs.

## Discussion

This WISN study results clearly indicate that public sector healthcare providers in Bangladesh, in general, are suffering from a very high workload pressure (seven out of 20 staff categories under study have WISN ratio between 0.30 and 0.49). The most overworked staff are the district hospitals consultants (most notably the Medicine, Pediatrics, Anesthesiology, Obstetrics and Gynecology, and Surgery Consultants). This also indicates the highest numbers of staff required are nurses and physicians at both DH and UpHCs.

Study results also indicate inadequacy of staffing norms in the public sector health facilities due to presence of high workload. Historically, Bangladesh has faced shortage of formally trained health workers. The country has thus been looking for innovative ways to achieve a more efficient use of its qualified health workforce in the health sector—particularly in the context of increased demand for health services, evolving disease management policies and demographic changes [10].

Even in facilities that are considered to be ‘well’ staffed, their staffing levels are not adequate to provide the full range of services required and to handle the accompanying workload. This clearly demonstrates the need to review the existing staffing norms of public sector health facilities with a view of equitably providing at least the essential service package in the country.

High workload pressure also has serious consequences, namely, fatigue and burnout of service providers, lack of motivation, and compromised quality of care [17]. High workload is, however, not unique to Bangladesh. WISN studies in low- and middle-income countries like Uganda [9], Burkina Faso [18], Iran [19], Kenya [20], and Namibia [21] also identified high workload pressure among their human resources for health.

The high workload of the staff at the district level and below have management implications for the delivery of quality ESP services. For example, due to high number of patients, staff are unable to provide services within the standard amount of time. This might be a coping mechanism of service providers to reach services to the maximum number of patients. The other side of the coin is that service providers may not be optimizing their working time, for example, by not coming on time or not staying the full official duty time. Nevertheless, contact time with the patient is an established predictor of quality of care [22], patient satisfaction [22, 23], and responsiveness [24]. A recent study from Bangladesh showed the outpatient department consultation time in public sector health facilities range from 48 s to 4.04 min per patient [25, 26], which is also low, as we found in this study. Results from this study are consistent with the findings of a similar WISN study conducted in other two districts in Bangladesh [27].

A culture of workload-based human resource management systems needs to be developed and this could be done through systematic planning and strong monitoring platforms. This might include establishing well-defined roles and responsibilities of the team responsible for WISN application—with clear deliverables, timelines and reporting structures. In Namibia and Uganda, WISN application taskforces directly report to the Permanent Secretary of the Health Ministry. The taskforce should be given adequate authority and autonomy to carry out the implementation.

This WISN study was utilized to facilitate a national policy dialogue on health workforce in April 2019 with participation of the most senior officials (Health Secretary and the Director General of health services) of the MOHFW, among others. The results of the study were used to advocate for the review of the current 2008 national staffing norms and for increasing the demand, availability and investment in the health workforce in Bangladesh. The results of this study have been used to support updating the staffing norms of the Table of Organization and Equipment of 50-bed, 250-bed and 500-bed hospitals in Bangladesh [28]. Increasing the investment on and the availability of health workers became even more relevant during the COVID-19 pandemic. Bangladesh went into the COVID-19 pandemic with a high shortage of health workers (especially doctors, nurses and laboratory workforces) and this has been exacerbated during the response to the pandemic by hampering not only the COVID-19 response, but also availability of essential health services [29].

### Strengths and limitations

Major strengths of this study were such as setting activity standard was supported by time-motion study which helped the expert groups to determine context specific activity standard. The study adopted both qualitative and quantitative methods for primary data collection, which complemented each other for bringing data accuracy. Technical inputs received from the WHO’s international technical experts, and officials of the MOHFW improved the data quality as they were directly involved in quality checks at the field level.

Despite careful planning and thorough implementation of the assessment, the study team faced some challenges. First, some service statistics data, which were essential for establishing standard workloads, were unavailable (e.g., OPD visits data for the DH Consultants, separate follow-up antenatal care data below the Upazila level, etc.). Second, the official number of existing staff often did not match with the number of staff the data collection team observed providing services (e.g., SACMOs, though they were posted at Union-level facilities, they were found to provide service at the UpHCs for 3 days a week). These often confused our data collectors and had to deal with these issues and resolved in consultation with respective heads of institutions during data collection.

### Recommendations

The findings of this study have several broad implications that can help guide HRH investments by the government as well as health and development partners. First, in the short term, reallocation of staff from low workload areas to high workload areas could be affected. For example, in Shailkupa UpHC, there are 15 SACMOs, with an excess supply of almost five. Workload is Low with a WISN ratio of 1.48. Conversely, in nearby Kotchandpur UpHC, there are seven SACMOs, with a shortage of − 6.81. Workload is High with WISN ratio of 0.51. At least five SACMOs from Shailkupa can be reallocated to Kotchandpur to tackle the high workload.

Second, the current scope of work of nurses should be changed to reduce their support services. As evidenced from the WISN results, nurses were the most needed staff, one of the most overloaded, and short in supply. On top of all these, they had been burdened with support activities. If some of their support and additional activities could be shifted to other staff, nurses could devote their time better in nursing care.

Third, task shifting could be adopted as a strategy to reduce consultants’ workload. Since the Consultants, especially those of Medicine, Pediatrics, Anesthesiology, Obstetrics and Gynecology, and Surgery were undergoing an extremely high workload pressure, their tasks might be shifted to other staff. General physicians, nurses, and midwives could be engaged in some of the tasks that consultants currently undertake. Since the government is currently developing the midwifery cadre and recruiting them in larger numbers, they might be engaged to perform some of the current functions performed by the Gynecology and Obstetrics Consultants such as conducting ANC, PNC, and new-born care [30].

In the long term, the government of Bangladesh should review and revise the current staffing norms based on health facility workload. Although in some cases, filling up vacant posts can improve the workload situation, in many others this is not much helpful. In district hospitals, even if all sanctioned posts are filled out for consultants, general physicians and nurses, still the WISN ratio would not be normal (i.e., between 0.90 and 1.19). Therefore, policy-makers should review the staffing norms of health facilities based on that facility’s workload and make decisions specific to the individual local context. To this front, the WISN method could be an objective tool to show locally appropriate staffing levels and help to achieve a more efficient use of scarce resources, while improving healthcare coverage across the district health system.

## Conclusion

The current staffing norms of public sector health facilities in Bangladesh appear not responsive to actual health needs and demand of the population in the selected districts where the WISN method was applied and likely to be the case in other parts of the country, given its centralized governance structure. A review of the national staffing norms will be critical in reducing shortages of health workers as high workload prevailed already prior to the COVID-19 outbreak. It now has become even more pressing, as workload has significantly increased, and more health workers are needed to effectively respond to COVID-19 and to maintain essential health services. With a vision of becoming an upper middle-income country by 2021 and meeting the commitment towards Universal Health Coverage by 2030, Bangladesh needs to optimize its human resources. Such assessments can aid policy and decision-makers in this direction.

## Data Availability

The datasets generated and/or analyzed during the current study are not publicly available due to joint ownership of the data, but they are available from the corresponding author on reasonable request. Data may be obtained from the World Health Organization Bangladesh Country Office on reasonable request (Focal Point: NPO-HRH: nuruzzamanm@who.int).

## Abbreviations

AWT: Available working time
CC: Community clinic
DH: District Hospital
ESP: Essential services package
EWGs: Expert working groups
FWV: Family welfare visitor
GOB: Government of Bangladesh
HRD: Human resources development
HRH: Human resources for health
IDIs: In-depth interviews
KIIs: Key informant interviews
MO: Medical officer
MCWC: Maternal and Child Welfare Center
MOHFW: Ministry of Health and Family Welfare
SACMO: Sub-Assistant Community Medical Officer
SC: Steering committee
TT: Technical taskforce
UHFWC: Union Health and Family Welfare Center
UpHC: Upazila Health Complex
USC: Union sub-center
WHO: World Health Organization
WISN: Workload indicators of staffing need
